# The Impact of Course Title and Instructor Gender on Student Perceptions and Interest in a Women's and Gender Studies Course

**DOI:** 10.1371/journal.pone.0106286

**Published:** 2014-09-30

**Authors:** Jennifer R. Spoor, Justin J. Lehmiller

**Affiliations:** 1 Department of Management, La Trobe Business School, La Trobe University, Melbourne, Victoria, Australia; 2 Department of Psychology, Harvard University, Cambridge, Massachusetts, United States of America; Georgetown University Medical Center, United States of America

## Abstract

Diversity awareness has enormous benefits, and universities in the United States increasingly require students to complete diversity-related courses. Prior research has demonstrated that students' initial attitudes toward these courses affect their subsequent engagement, as well as the quality of their learning experience; however, very little research has examined how these initial attitudes are formed. We conducted an experiment to examine this issue in the context of a women's and gender studies course in psychology. Participants read one of two identical course descriptions that varied only the course title (i.e., Psychology of Gender versus Psychology of Women) and instructor gender. Participants perceived a women-titled course to be narrowly focused compared to an identical gender-titled course and were more interested in taking the gender-titled course. Instructor gender had no effects on any of the variables. Additionally, female participants had more positive attitudes toward the course than male participants, regardless of title. Exploratory mediation analyses indicated that the main effects of course title and participant gender were mediated by perceptions of course content. Implications for improving student experiences and interest in diversity-related courses are discussed.

## Introduction

Given the increasingly diverse workforce and student population, there has been a substantial push to increase diversity awareness in higher education [1]. Many universities have added diversity-related requirements to some of their degree programs [2], and there have been calls for wider implementation of diversity education requirements [3]. There is a large body of research in both academic and workplace settings examining how diversity training influences attitudes towards diversity, knowledge and skills [4]. Within academic settings, research suggests that these courses provide numerous benefits to students [5]–[7], especially if students already have favorable attitudes toward the subject [8]. However, while prior research demonstrates that students' initial attitudes toward their diversity-related course influence their subsequent engagement with the class and the quality of their learning experience, relatively little research has directly examined the factors that shape those initial attitudes. The present research examines two contextual and one student demographic factor that may affect prospective students' initial interest in and expectations for a diversity course related to gender: course title, gender of the instructor, and gender of the student.

### Attitudes toward Women's and Gender Studies Courses

Students who take women's and gender studies (WGS) courses reap many benefits, such as increased egalitarian attitudes [6], [9], higher achievement goals and professional confidence [7], [10], and improved cognitive development [11]. Importantly, both women and men benefit from these courses [9], [12]. While most students in WGS courses report satisfaction [13], there are notable exceptions. Professors of WGS courses frequently report anecdotes of dissatisfied and highly resistant students [14], [15], and research using end-of-semester evaluations suggests that students are more likely to describe WGS instructors as biased and unreasonable compared to other instructors [16]. Importantly, students who begin these courses with resistant attitudes and negative expectations tend to be less engaged and report more negative experiences during the subject [8], [13], though positive change is still possible [8], [17].

Despite evidence that some students are resistant toward WGS courses, very little research has examined the source(s) of this negativity [13]. Indeed, most research on WGS courses examines outcomes among students who have already self-selected to take a WGS course, and though some studies have utilized carefully selected comparison groups [17], [18], most prior research does not account for why students may opt out of taking a WGS course in the first place. One exception is evidence from prior research suggesting that students who select WGS courses tend to have more egalitarian attitudes compared to those who select other classes [19], [20]. Thus, more research is needed to understand the factors that shape students' attitudes and interest in WGS courses, and experimental research that minimizes self-selection biases is essential. The current research focuses on two contextual factors, course title and instructor gender, because of their potential broad impact and because their effects can be addressed in pedagogical and administrative decisions. These factors are experimentally manipulated using random assignment to examine their causal influence and control for pre-existing attitudes. The current research also examines student gender because while the subject matter is highly relevant to both women and men [21], WGS instructors often note that relatively few men enroll in the their courses [22].

### The Importance of Course Title and Instructor Gender on Course Expectations

The current research examines how students' initial attitudes toward a WGS course are shaped before they decide to enroll in a course. While attitudes can develop via in-depth, systematic processing, heuristics and other cues can both directly affect attitudes and bias how attitude-relevant information is processed [23], [24]. Though cues may provide information with limited diagnostic value, prior research demonstrates that people are quick to form attitudes and impressions based upon limited information [25], [26] and are often poor at correcting their initial impressions upon learning new information [27]. The current research focuses on two contextual cues that are readily available to students prior to enrollment and that may bias how they think about the course: course title and instructor gender.

Although the specific title may differ, psychology WGS course titles typically reference either women or gender [22]. It would make intuitive sense that different titles might be perceived differently by students; however, research has yet to address whether and how course title affects perceptions of a WGS course. Certainly, the content and goals of courses titled Psychology of Women versus Psychology of Gender probably should differ (and sometimes do), but this may not always be the case. For example, faculty members may be assigned to teach a class with a specific title but prefer to focus on different content, and some course titles reflect past curriculum decisions that did not account for how the course title would be perceived by students or how the course would actually be taught. Further, many departments do not offer both versions of the course, which may contribute to further overlap in their content because instructors may feel that the course has to serve multiple purposes, irrespective of title.

A course titled Psychology of Women is likely to be perceived to focus more strongly on women's issues, whereas a course titled Psychology of Gender may be perceived to focus more broadly on women and men. While these different perceptions may reflect real differences as noted above, some research suggests that courses that appear to focus on a traditionally disadvantaged group might be perceived negatively and thus be of less interest to some students, regardless of actual content. For example, research in the context of workplace training suggests that the term “diversity” is often perceived to be narrowly focused on race and gender issues, and many organizations prefer broader terms to describe their diversity training programs (e.g., “Valuing Differences”) in an attempt to increase engagement among staff [28]. Indeed research on how diversity training is framed suggests prospective trainees use a variety of cues to infer what the course will be like and that diversity training that is perceived to be more broadly focused is often evaluated more favorably than comparable training that is perceived to be narrowly focused [28], [29].

Within the context of WGS courses, some students may perceive that a class focused on women and women's issues is irrelevant and outdated. Research on perceptions of gender inequality has shown that many people believe that gender inequality and discrimination have decreased over time [30], [31]. Given these more general beliefs about gender inequality, it is not surprising that some students report that WGS content is irrelevant or unimportant to them [32]. Students may also use the course title to infer whether the course will focus on feminism. Some WGS instructors and courses do focus on feminism [21], [33], so there may be some kernel of truth to this inference, but a real or perceived focus on feminism may be unappealing to some students. Despite the overall positive impact of the various strands of feminism, negative stereotypes of feminism and feminists persist [34]–[36]. Many students hesitate to identify themselves as feminists [18], [37], even when their personal beliefs align with feminist values [37], [38] and when they hold positive implicit associations with feminism [39]. Thus, if students perceive that a WGS course has narrow content that emphasizes feminism, they may be less favorable toward the course and less willing to enroll.

In addition to course title, a second piece of information that is often readily available to students is the instructor's gender. While traditionally taught by women, WGS courses are increasingly taught by male instructors [12], which may affect prospective students' expectations for the course. Research suggests that students tend to rate male instructors more favorably than female instructors [40], [41], though this effect is attenuated in the humanities and social sciences [41]. In the context of a WGS course, female instructors may be perceived as credible due to their perceived expertise in this area, but source credibility also tends to decrease when sources argue in their self-interest [42]. WGS classes are frequently perceived to focus on gender inequality in ways largely perpetrated by men [12], thus male instructors might be perceived as less self-interested and thus more credible than female instructors. Indeed, research suggests that students expect male teachers of WGS courses to be highly credible [43]. These expectations for credibility subsequently affect how the WGS message is evaluated, such that students rated a lecture on gender inequality as more accurate when it was delivered by a male rather than by a female professor [44]. There is some evidence that biased evaluations of male and female professors may begin before any actual course content is delivered. For example, in a study of perceptions of a *Sociology of Gender* course, participants expected a female instructor to include more biased and political content compared to a male instructor [45]. This difference emerged despite the fact that participants read an identical one-page syllabus that only varied the instructor gender (not course title). Thus, students may be more favorable to a WGS course taught by a man rather than by a woman. Because the “Psychology of Women” title may signal that the class will focus on women, the different evaluations of the male and female instructor may be stronger when the course title mentions women compared to when it mentions gender.

### The Importance of Student Gender on Course Expectations

The majority of students in WGS courses are women [22], indicating that women are generally more favorable toward and interested in WGS courses, regardless of title or instructor gender. However, because the subject is highly relevant to both women and men [21], and because WGS instructors often cite the low enrollment of male students as a potential negative aspect of teaching such courses [22], it is important to examine how course title and instructor gender affect how women and men perceive the course. One possibility is that male students will be even more strongly affected by the course title and instructor gender cues compared to female students (i.e., recipient effects) [24]. As noted above, some students believe that WGS content is irrelevant or unimportant to them [32], and this belief may be held more strongly by men. As a traditionally high status group, men may be motivated to protect the status quo [46] and thus may evaluate negatively courses and initiatives that they think will challenge their privileged position [47]. Additionally, men may avoid WGS courses that are perceived to have feminist content because men are more likely than women to have negative implicit associations with feminism [39]. Thus, male students may be more attracted to a WGS course that emphasizes gender than one that emphasizes women.

Participant gender may also affect reactions to instructor gender, such that men may be more likely than women to prefer instructors of the same gender. For example, research on end-of-semester evaluations suggests that whereas female students tend to give comparable teaching evaluations to their male and female instructors, male students tend to evaluate their male instructors more favorably than their female instructors [40], [41]. Thus, male students may be more attracted to a WGS course ostensibly taught by a man. To explore these possibilities, participant gender was included as a predictor variable.

### Overview of the Current Research

The current research examined prospective students' attitudes toward a WGS course, depending upon course title, instructor gender, and students' own gender. Participants were given an identical description of a course titled either Psychology of Women or Psychology of Gender. Instructor gender was also varied via the name of the ostensible instructor. Evaluations of the course were conceptualized in terms of perceptions of the course content and willingness to enroll.

Based on the literature reviewed above, we expected three main effects. First, we expected a main effect for course title, such that the Gender course would be evaluated more favorably and be perceived as more broadly focused compared to the identically described Women course (Hypothesis 1). We also expected a main effect for instructor gender, such that the male instructor would be evaluated more favorably and as more broadly focused compared to the female instructor (Hypothesis 2). We also expected that participant gender would affect perceptions of the course, such that women would evaluate both WGS courses more favorably compared to men (Hypothesis 3).

We also examined three potential interactions. First, we explored a course title by instructor gender interaction, whereby the different evaluations of male and female instructors would be stronger in the Women course compared to the Gender course. We also explored whether men would be more interested in a Gender course compared to a Women course (i.e., course title by participant gender interaction) and whether men would be more interested in taking the course from a male instructor compared to a female instructor (i.e., instructor gender by participant gender interaction).

Prior research suggests that general attitudes can affect interest in enrolling in specific courses [19], [20]. This suggests that more specific attitudes toward a WGS course, which we hypothesize are affected by course title, instructor gender, and participant gender, may in turn influence willingness to enroll in the course (i.e., attitudes toward the course are the mediator). Thus, we also explored whether the effects of the predictor variables on willingness to enroll would be mediated by perceptions of the course content.

## Method

### Ethics Statement

The Institutional Review Board of Colorado State University approved the procedures for the experiment. Informed consent was obtained via a written consent form provided at the beginning of the experiment.

### Participants and Procedure

Participants were 352 introductory psychology students (218 women, 134 men; *M*_age_ = 19.26, *SD*_age_ = 2.03, Range = 17 to 35) who participated in exchange for partial course credit. The majority (84.4%) of participants self-identified as European American.

Participants were randomly assigned to condition in a 2 (course title)×2 (instructor gender) between-participants design.

Participants signed up to complete a study of perceptions of college-level courses. After providing informed consent, participants then read that the study was about students' perceptions of potential college courses and that they would read about one or more college-level courses. All participants then read a short description of a psychology WGS course, completed the dependent measures, and were debriefed and thanked. Participants also completed additional measures that were included for exploratory purposes and are not discussed further. Copies of the full questionnaire are available from the first author.

### Materials

Participants were shown a description of a WGS psychology course. The course title and instructor gender (the independent variables) were listed at the top of the page. The course title either emphasized the traditionally disadvantaged group (Psychology of Women) or all genders (Psychology of Gender). The instructor's gender was manipulated through either a male (William Smith) or female (Wendy Smith) name.

The course title and instructor gender information was followed by a brief course description that was based on one previously used for both a Psychology of Gender and a Psychology of Women course taught by the first author. Because the course description is typically available to prospective students, we included it to increase mundane realism. Further, providing additional, albeit minimal, information increases people's feeling that they are entitled to make a social judgment [48]. The additional information described the course as focused on social science research related to gender and mentioned both women and men. The additional information is provided below:

This course will introduce you to the scientific literature on gender and the psychology of gender as approached from the perspective of a social scientist. One emphasis is on gender stereotypes vs. actual gender differences in abilities, personality, and social behavior and the possible causes of such gender differences. The implications of gender roles for the behavior of women and men will be examined through detailed study of social behaviors. Basic and applied research on topics such as close relationships, work, sexual harassment, and violence will also be reviewed. The format for the class is primarily lecture but will also include class discussion, activities in small groups, video presentations, and guest lectures. You are responsible for all announcements and information provided in class.

We conducted a pre-test in which introductory psychology students (*N* = 62) were given materials identical to the current experiment and asked to complete similar questionnaire items, as well as memory checks for the manipulations. Analysis of the memory checks indicated that the manipulations were successful. Participants in the pre-test who were shown the ‘psychology of women' title were more likely to agree that the course had been titled Psychology of Women (and less likely to agree that it had been titled Psychology of Gender) than participants in the ‘psychology of gender’ condition, *F*(1, 55) = 64.90, *p*<.001. Participants in the female instructor condition were more likely to agree that the instructor had been female than participants in the male instructor condition, *F*(1, 55) = 44.42, *p*<.001.

### Measures

All measures were assessed on 7-point scales anchored at 1 (*strongly disagree*), 4 (*neither agree nor disagree*), and 7 (*strongly agree*).

#### General perceptions of the course

Four items assessed general perceptions of the course and were intended to reinforce the cover story and verify that the manipulations only affected perceptions of course content. The general course perception items were adapted from questions that typically appear on end-of-semester course evaluations at this university. Participants indicated their agreement that “the goals of this course are clearly stated”; “the instructor appears well-organized”; “the instructor appears available to students”; and “the requirements for this course appear to require a reasonable amount of work”. Responses were averaged into a composite measure of general course perceptions (α = .70).

#### Perceptions of course content/focus and credibility

Four items assessed participants' perceptions of whether the course content was likely to focus on women's issues and feminism. Participants indicated the extent to which they thought the course would focus “equally on women and men's issues” and “primarily on women's issues”. Participants also responded to two items specifically assessing perceptions of feminist content: “This course looks like it will be about feminism” and “This course looks like it will be influenced by feminism”.

Course credibility was assessed with three items inquiring whether the course appeared comprehensive and the instructor seemed credible. The items were: “The instructor will probably provide a fair and balanced perspective on these topics”, “The instructor appears to be credible”, and “Overall, this course looks like it is comprehensive”.

These seven items were submitted to an exploratory factor analysis with principal axis factoring and promax rotation. This analysis yielded two factors, accounting for 56.83% of the variance; all items had loadings greater than .60 on one factor and lower than .25 on the other. Inspection of the pattern matrix revealed that the four items assessing course focus (i.e., equal focus, focus on women, focus on feminism, influenced by feminism) loaded on the first factor. A composite measure consisting of these four items was created (α = .85) and scored such that higher scores indicated greater course focus on women and feminism. The three items assessing perceived credibility (i.e., fair and balanced perspective, credible, comprehensive) loaded on the second factor. A composite measure of these items was created (α = .73), and scored such that higher scores indicated higher credibility and less bias.

**Willingness to enroll in the course.** Finally, participants indicated their interest in taking the course with three items: “Overall, this course looks like a course I would want to take”, “If this course were offered at my university, I would be willing to sign up for it”, and “I would enjoy taking this course”. Responses were averaged into a composite measure (α = .94).

## Results

Data files are available from the first author upon request. The dependent measures were submitted to separate 2 (course title)×2 (instructor gender)×2 (participant gender) ANOVAs.

### General Perceptions of the Course

As expected, participants' general perceptions of course content were not affected by course title, instructor gender, or participant gender as there were no statistically significant main effects or interactions, *F*s<3.73, *p*s>.06, η_p_^2^s<.012.

### Perceptions of Course Focus and Credibility

For course focus, there was a significant main effect for course title, *F*(1, 344) = 63.86, *p*<.001, η_p_^2^ = .16, as well as a significant main effect for participant gender, *F*(1, 344) = 5.14, *p*<.03, η_p_^2^ = .02. Consistent with Hypothesis 1, participants perceived the women-titled course to be more focused on women and feminism (*M* = 4.71, *SE* = 0.10) than the gender-titled course (*M* = 3.54, *SE* = 0.11). Consistent with Hypothesis 3, male participants perceived all courses to be more focused on women and feminism (*M* = 4.29, *SE* = 0.12) than female participants (*M* = 3.96, *SE* = 0.09). Neither the main effect of instructor gender nor any of the two- or three-way interaction terms were significant, *F*s<2.12, *p*s>.14, *p*s>.07, η_p_^2^s<.007.

Similarly, for course credibility, the main effect for course title was significant, *F*(1, 344) = 8.55, *p*<.01, η_p_^2^ = .02, as was the main effect of participant gender, *F*(1, 344) = 9.40, *p*<.01, η_p_^2^ = .03. Consistent with Hypothesis 1, participants rated the gender-titled course higher in credibility (*M* = 4.84, *SE* = 0.10) than the identical women-titled course (*M* = 4.45, *SE* = 0.09). Consistent with Hypothesis 3, female participants (*M* = 4.85, *SE* = 0.08) rated all courses higher in credibility than did male participants (*M* = 4.41, *SE* = 0.11). No other significant main effects or interactions emerged, *F*s<3.30, *p*s>.07, η_p_^2^s<.01.

Thus, participants assumed that the course titled Psychology of Women would be more focused on women and feminism, and also that this course would have less credibility, compared to an identical Psychology of Gender course. This occurred even though the course descriptions were identical and described both women and men in the context of a course focused on empirical research.

### Willingness to Enroll in the Course

The manipulations also affected participants' interest in taking the course. The ANOVA revealed significant main effects for course title, *F*(1, 339) = 10.19, *p*<.01, η_p_^2^ = .03, and participant gender, *F*(1, 339) = 33.51, *p*<.001, η_p_^2^ = .09. Participants expressed more interest in taking the gender-titled course (*M* = 4.56, *SE* = 0.12) than the women-titled course (*M* = 4.02, *SE* = 0.12), and female participants were more interested in taking either course (*M* = 4.78, *SE* = 0.10) than male participants (*M* = 3.80, *SE* = 0.13). Neither the main effect for instructor gender nor any of the interaction terms was significant, *F*s<1.13, *p*s>.28, η_p_^2^s<.004. These results point to a potentially worrisome effect in that course title may impact who actually ends up enrolling in the class, such that the gender title may appeal to a broader number of students than the women title.

### Mediation Analyses

Though the central aim of this research was to examine the direct effects of our predictor variables, we wanted to explore whether course title and participant gender affected willingness to enroll in the course through changes in perceptions of course focus and course credibility (i.e., mediation). We used multiple-mediator regression models [49] to test simultaneous mediation.

The following conditions provide evidence for simple mediation and can be extended to the multiple mediator case [49], [50]: (1) the independent variable should significantly predict both the mediator and the dependent variable, (2) the mediator should significantly predict the dependent variable, and (3) the relationship between the independent and dependent variables should be reduced when the mediator is also included in the model. To test whether perceived course content and instructor credibility served as mediators simultaneously, we estimated a multiple-mediator regression model using the SPSS macro developed by Preacher and Hayes [49]. We conducted these analyses separately for the relationship between course title and willingness to enroll and the relationship between participant gender and willingness to enroll.

To examine multiple mediation of the relationship between course title and willingness to enroll, course title was the dummy coded (0 =  gender title, 1 =  women title) predictor variable. Instructor gender and participant gender were also dummy coded (0 =  male, 1 =  female) and included as control variables. Course title significantly predicted both mediators: course focus, *B* = 1.18, *SE* = .14, *t* = 8.35, *p*<.001; course credibility, *B* = −.35, *SE* = .13, *t* = −2.74, *p*<.007. Course title also significantly predicted the dependent variable willingness to enroll, *B* = −.50, *SE* = .16, *t* = −3.11, *p* = .002; and both mediators predicted willingness to enroll: course focus, *B* = −.34, *SE* = .05, *t* = −6.21, *p*<.001; course credibility, *B* = .47, *SE* = .06, *t* = 7.70, *p*<.001. When the independent variable and both mediators were included in the model predicting the dependent variable, the relationship between course title and willingness to enroll became non-significant, *B* = .06, *SE* = .15, *t*<1. To test whether the multiple mediation was significant, we used Preacher and Hayes' [49] bootstrapping macro for SPSS with 5,000 bootstrapped re-samples to estimate bias-corrected and accelerated 95% confidence intervals for the total indirect effect (i.e., including both mediators), as well as confidence intervals for the specific indirect effect of each mediator controlling for the presence of the other mediator. Confidence intervals that do not include zero provide evidence for mediation. None of the confidence intervals for the indirect effects included zero (total indirect effect: −.77 to −.38; course focus: −.58 to −.26; course credibility: −.31 to −.05).

To examine multiple mediation of the relationship between participant gender and willingness to enroll, participant gender was the dummy coded predictor variable, and instructor gender and course title were included as dummy coded control variables. Participant gender significantly predicted both mediators: course focus, *B* = −.33, *SE* = .15, *t* = −2.31, *p*<.03; course credibility, *B* = .39, *SE* = .13, *t* = 3.05, *p*<.003. Participant gender also significantly predicted the dependent variable, willingness to enroll, *B* = .98, *SE* = .17, *t* = 5.91, *p*<.001. When the independent variable and both mediators were included in the model predicting the dependent variable, the relationship between course title and willingness to enroll was reduced in magnitude but was still significant, *B* = .68, *SE* = .15, *t* = 4.63, *p*<.001. Bootstrapping analyses confirmed mediation, given that none of the bias-corrected and accelerated 95% confidence intervals for the indirect effect contained zero (total indirect effect: .14 to .47; course focus: .03 to .23; course credibility: .07 to .32).

These results provide evidence that the relationship between course title and willingness to enroll was simultaneously mediated by both perceptions of course content and perceptions of instructor credibility. Thus, reduced interest in taking the Psychology of Women course reflected perceptions that this course would be more focused on women and feminism, and that the course would be lower in credibility, compared to the Psychology of Gender course. In addition, female participants' greater interest in taking both courses reflected their perception that the courses would be less focused on women and feminism, as well as higher in credibility, compared to male participants.

### Ancillary Analyses

We examined whether participants' prior knowledge of a psychology WGS course offered at their university affected responses to the manipulations. Only four participants indicated having previously taken a WGS course (i.e., Women/Gender in Society or Introduction to Women's Studies). Moreover, although over one-third of the sample (*n* = 137) indicated that they were aware their university offered a similar WGS course, participants reported knowing very little about the course itself (*M* = 2.98 on a 7-point scale). Adding the dichotomous question regarding awareness of the psychology WGS course as an independent variable to the ANOVAs yielded a similar pattern of results as reported above, and there were no consistent effects for awareness on the dependent measures.

## Discussion

These results indicate that in the context of WGS courses, both course title and participant gender contribute to the expectations students have regarding the course. Consistent with Hypothesis 1, participants expected the course to have broader coverage and be higher in credibility when it had the more inclusive gender title than an identical course where the title focused on the traditionally disadvantaged group, women. Consistent with Hypothesis 3, female participants perceived both courses to have broader coverage and to be higher in credibility compared to male participants. Furthermore, participants' interest in taking the course was independently affected by both course title and participant gender, such that participants were more interested in taking the gender-titled course than the women-titled course, and female participants were more interested than male participants in taking either course. There were no main effects for instructor gender on any of the dependent variables, thus Hypothesis 2 that male instructors would be perceived more favorably than female instructors was not supported. Furthermore there were no interaction effects; thus our exploratory hypotheses of moderation (i.e., moderation of course title effects by either instructor or participant gender and moderation of instructor gender effects by participant gender) were not supported. However, mediation analyses did suggest that differences in participants' willingness to enroll in the course depending on course title and participant gender were partly explained by how these variables affected perceptions of course content and credibility.

It is important to note that more general perceptions of the course, such as whether the instructor was organized and available to students, were not affected by the manipulations. Thus, the findings in the current research seem to reflect students' reactions to their perceptions of gender issues and feminism, and not a general negativity to university courses. Indeed, Hartung [16] suggested that WGS instructors may receive negative evaluations “based on who the students perceive [s/he] is rather than how [s/he] teaches” (p. 262). The current research suggests that students develop these perceptions early in their exposure to a WGS course. Though the finding that students develop negative perceptions of certain WGS courses quite quickly is unfortunate, the results are consistent with anecdotes from WGS instructors who frequently encounter resistant students who perceive them as biased [14], [15]. Such negative impressions are a barrier to achieving gender equality, and greater knowledge of this barrier can assist in breaking it down. WGS instructors might directly benefit from greater awareness of the sources of student resistance [29].

The differences that emerged for participant gender are consistent with the overall enrollment trends within WGS courses, such that women appear more motivated to take these classes. The fact that participant gender did not interact with either instructor gender or course title suggests that women and men respond similarly to these differences, but men may have a higher level of initial resistance. Future research might examine ways to increase men's motivation and interest in taking WGS courses.

Finally, it is interesting that we did not obtain any effects for instructor gender, which contradicts some earlier findings [45]. This difference could reflect the fact that with the increasing number of women teaching in university settings, especially in psychology departments, instructor gender is less distinctive as a cue. The lack of an effect should be encouraging to both women and men who teach WGS courses, because the current studies suggest that students do not assume that either gender is more capable or less biased regarding the subject. Thus, men might be encouraged to teach such classes, and women may feel empowered that they do not need to “prove” their credibility, though we note that WGS instructors may still face resistance once in the classroom [12].

### Strengths and Limitations

The current research used an experimental design to examine how course title, instructor gender and student gender affect students' perceptions of a WGS course prior to their actual exposure to the course. The experimental nature of the research increases our confidence that the course title itself biased participants' course perceptions. The use of the experimental design also helps rule out self-selection biases that are inherent when surveying students who have already enrolled in their courses.

We do note a few limitations and directions for future research. First, we examined reactions to a WGS course in the context of a psychology department, thus it is possible that the effects do not extend to WGS courses taught in other departments (e.g., history, women's studies). Though previous research has examined the role of instructor gender in expectations for diversity courses taught in other disciplines [45], [51], those studies did not simultaneously vary the focus of the course title, so future research might benefit from examining a wider range of WGS courses and titles. We also note that the course description in the current studies emphasized empirical social science research regarding both women and men, whereas some WGS course descriptions might emphasize women's experiences or critical discourse, which may appear more subjective to prospective students. From our perspective, the fact that we obtained effects even when the course was described as having relatively objective and inclusive goals indicates that students' biased perceptions of WGS courses might be quite pervasive. However, by taking an even-handed tone, our course description may have obscured potential effects. Future research might examine a range of course descriptions.

The current research also focused on WGS courses, but the range of diversity course offerings is quite broad, including courses in Black or African American studies, Hispanic studies, Aboriginal studies, Queer studies, or more general diversity content. The current research suggests that initial interest and reactions to these courses might also be affected by the extent to which the course title appears inclusive or focuses on the disadvantaged group, as well as participants' socio-demographic characteristics. Additional research examining these factors in relation to other diversity courses and other socio-demographic characteristics (e.g., ethnicity, sexual orientation) is needed. Similarly, although we found no effects for instructor gender, other instructor characteristics, such as sexual orientation or ethnic background, may contribute to students' early perceptions of a diversity course [52], [53]. Future research might use qualitative approaches, such as interviews or focus groups with prospective students, to uncover the additional factors that may be important to explore in future experimental research.

### Implications and Conclusions

Higher education is one arena in which students can learn about inequality and explore new ways of thinking about gender and other majority/minority relations. In the context of women's and gender studies, the results of the current research suggest that both women and men should play a role in delivering this curriculum. Importantly, the current research suggests that students might be equally receptive to men and women teaching such courses, which might help encourage men to be more involved in this area. These results also suggest that there may be benefits to naming a WGS course with a broader, more inclusive title. However, we are not suggesting that WGS courses (or other diversity-oriented courses) should always be named with a more inclusive title. Diversity courses provide a voice for underrepresented and disadvantaged groups, and help provide balance and equity in university curricula. Thus, diversity courses often intentionally place minority groups' experiences front-and-center and are purposefully unbalanced in focus. While course titles should accurately reflect the course's focus and goals, the current research suggests that consideration might also be given to the issue of how to attract a wide range of students who will approach diversity classes with positive expectations, which may increase the likelihood that they will benefit from exposure to diverse perspectives.
